# The effect of Caspian Sea water on mechanical properties and durability of concrete containing rice husk ash, nano ${\varvec{\hbox {SiO}_2}}$, and nano ${\varvec{{\hbox {Al}}_{2}{\hbox {O}}_{3}}}$

**DOI:** 10.1038/s41598-022-24304-4

**Published:** 2022-11-23

**Authors:** Omolbanin Arasteh-Khoshbin, Seyed Morteza Seyedpour, Tim Ricken

**Affiliations:** 1 Institute of Structural Mechanics and Dynamics in Aerospace Engineering, Faculty of Aerospace Engineering and Geodesy, University of Stuttgart, Pfaffenwaldring 27, 70569 Stuttgart, Germany; 2 Porous Media Lab, Institute of Structural Mechanics and Dynamics in Aerospace Engineering, Faculty of Aerospace Engineering and Geodesy, University of Stuttgart, Pfaffenwaldring 27, 70569 Stuttgart, Germany

**Keywords:** Civil engineering, Structural materials

## Abstract

Various studies have been recently conducted aiming at developing more sustainable cementitious systems so that concrete structures may not have a negative effect on the environment and are decomposed. It has been attempted to build sustainable binders by substituting silica fume, cement with fly ash, nano-silica, nano-alumina, and rice husk ash. In this paper, a series of experiments on concrete with different contents of rice husk ash (10%, 15%, and 20%), nano${{\hbox {SiO}_2}}$ (1%, 2%, 3%, 4%, 5%, 6%, 7%, 8%), and nano$\hbox {Al}_{2}{\hbox {O}}_{3}$ (1%, 2%, 3%, 4%) are performed to analyze the durability and mechanical properties of samples under the curing condition of Caspian seawater. The workability, density, water penetration, chloride ion penetration, and compressive strength (at 7, 14, 28, and 90 day) of the samples were determined. The experimental results showed that workability decreased gradually with increasing additives content, while the compressive gradually increased. Among the additives, adding 8% of the nano${{\hbox {SiO}_2}}$ had the most significant effect on the improvement of compressive strength. Adding 8% nano${{\hbox {SiO}_2}}$ and 4% nano$\hbox {Al}_{2}{\hbox {O}}_{3}$ reduced the depth of water permeability by 53% and 30%, respectively. Furthermore, adding 8% nano${{\hbox {SiO}_2}}$ reduced chloride ion penetration by 85%.

## Introduction

The annual growth rate of the current world population is 1%, and it is estimated to reach 8.5 billion by 2030^1^. Consequently, the population is expected to increase to 9.8 billion by 2050^2^. It is needed to annually build 10–30 million new residences for accommodating the growing population. Besides, other infrastructural advances are required^3^. As a result of the growing demand for building, cement concrete construction is also increased, and it is estimated that the worldwide cement production rise by 2.1% annually by 2030, and it would reach a total of 1.7 times the amount that is currently produced^1^. The cement industry is an energy-intensive industry^4,5^ with a considerable carbon mark that threatens environmental sustainability^6^. The annual emissions of concrete industry is up to 2.8 Gt $\hbox {CO}_{2}$^2,7^, and it is estimated that concrete production accounts for 9% of overall greenhouse emissions^8^, and it is predicted that 9% of overall greenhouse emissions is originated from concrete production, and cement plants account for 7–8% of carbon dioxide emissions worldwide^9^. The two approaches for addressing the impact of the greenhouse gas from the cement industry are as follows: improving the cement production process efficiency, and partial replacement of normal cement with supplementary cementitious materials (SCMs). There are different SCMs for decreasing carbon dioxide release and improving the durability and mechanical characteristics of concrete, which include as follows: ground-granulated blast furnace slag^10^, silica fume^11^, calcined clays^12^, nano clay^13^, natural pozzolans^14^, carbon nanotubes^15^, graphene oxide^16^, nano$\hbox {TiO}_2$^17^, nano$\hbox {ZnO}_2$^18^, nano$\hbox {CaCO}_3$^19^, nano$\hbox {Fe}_{2}{\hbox {O}}_{3}$^20^, fly ash^21,22^, nano$\hbox {SiO}_2$ (NS)^23,24^, nano$\hbox {Al}_{2}{\hbox {O}}_{3}$ (NA)^25^, and rice husk ash^26,27^.

Rice husk is produced as a byproduct in areas where rice grows. For producing rice husk ash, rice husk burns at temperatures between 300 and 450 °C. 1000 kg of rice grain provides almost 200 kg rice husk. After burning, about 40 kg RHA are obtained, which indicates that almost 20% of ash is made from rice husk^28^, and above 75% of its weight is silica^29^. RHA can be potentially used as partial replacement for Portland cement for its non-crystalline silica content^30^. There are significant pozzolanic characteristics in RHA because of amorphous silica, contributing to its specific surface area and fineness^31^. The rice husk ash should have a particle size below 8 $\upmu {\hbox {m}}$ so that maximum pozzolanic properties can be obtained^32^. Using RHA, the concrete cost can be minimized^33^, the concrete’s mechanical properties^34,35^ and durability can be improved^28^. Besides, resistance to chemical attacks^36^ and corrosion of reinforced concrete can be enhanced by using RHA in concrete^37^.

In addition to waste concrete additives like RHA, nanomaterials are highly important in enhancing the durability and mechanical properties of concrete^38,39^. The concrete mixture can have an excellent performance because of the high-density continuous packing of binder constituents. Fluidity can be increased by the use of nano-additives in mixed design^40^. Among nano-additive for concrete, NA and NS have shown high efficacy to improve flexibility, durability, and strength^40–42^.

NS is a fluffy white powder that includes amorphous silica powder. NS has strong surface adsorption, a large specific surface area, great chemical purity, acceptable dispersion, and big surface energy for its small particle size^40^. Since the hydrophilic NS is highly dispersed in water, NS that is used in concrete is chiefly hydrophilic^43^. NS can serve as a nano-filler and fill the empty spaces between calcium-silicate-hydrate (C-S-H) gel particles. Besides, NS is a pozzolan that has a high rate of pozzolanic reaction for its high surface area to volume ratio, which implies considerable chemical activities. When an optimal amount of NS is incorporated into the concrete, it can increase the compressive strength and refine the pores. Consequently, the concrete water permeability is reduced^44^. Although any dose above the optimal level causes enhancing compressive strength relative to the control mixture, it does not rise it by more than the optimal replacement level^45^. With addition of 1 and 2% NS, it is possible to improve the concrete’s tensile strength by up to 17 and 24% compared to the control mixture^46^. Using the filler and pozzolanic features of NS, concrete microstructure quality can be improved by declining porosity and improving pore structure^40^. Hydraulic permeability of porous concrete is declined by a 5% replacement of cement with NS^47^. As demonstrated by Rapid Chloride Penetration Test, substitution of 3 and 6% of NS can considerably improve permeability reduction in concrete^5^. As revealed by the measurements of viscosity, amount of water needed for maintaining the same workability was increased by the addition of NS^40^. NS concentrations beyond a specific threshold resulted in higher autogenous shrinkage due to self-desiccation, leading to a higher breaking potential^48^.

Apart from NS, the NA impact on durability and mechanical characteristics of concrete were investigated^42,49^. NA is an amphoteric oxide powder with acid-base characteristics, a large specific area, and thermal and mechanical stability. C-A-S (calcium aluminum silicate) gel in concrete is improved using NA as a partial replacement by cement. There is an interaction between calcium hydroxide made by the hydration of calcium aluminates and nano-alumina. With the addition of NA, the water absorption, microstructure, and resistivity of cement-based materials can be enhanced^42,50^. With the addition of 0.2% by weight of NA, the concrete’s strength and deformation performance can be significantly improved^51^. It has been revealed that NA significantly elevates the elasticity modules (up to 143% at a dose of 5%). However, it modestly affects compressive strength^52^. Additionally, the overall porosity of NA can be reduced with the addition of NA^25^. The addition of NA reduced the concrete workability^53^. The frost resistance of concrete with NA is significantly enhanced because of a more compacted microstructure^54^.

The matrix of concrete constructions should be taken into account to increase their durability. The interface transition zone between the coarse aggregate and mortar matrix is often the weakest link of conventional concrete. With the addition of RHA and nano partials like as NS and NA to the admixture, this weakness may be addressed, consequently enhancing the concrete’s strength. Mechanisms of these SCMs on concrete may be related to the fact that they function as Micro or Nano-sized fillers to reduce the Micro or Nano-sized pores in the hydration products of the cementitious materials and encourage the formation of a high-density C–S–H phase^55^. Despite the large number of studies investigating the role of NS, NA, and RHA in changing the durability and mechanical properties of concrete, only a few number of comparative studies have investigated the effects of all of them on concrete properties. Moreover, most works have just focused on studying normal curing conditions. Moisture and minerals cause significant concrete corrosion on beaches, which destroys inner bars^56^. Thus, the lifespan of concrete constructions in coastal environments is much shorter than elsewhere^57^. The impact of seawater on concrete is notable for many considerations. First, coastal and offshore constructions are subjected to both physical and chemical deterioration at the same time^58^, and second, oceans cover 80% of the earth’s surface^57^. The widespread use of concrete piers, breakwater decks, and retaining walls in the building of ports and docks. Consequently, it is important to investigate the mechanical properties and durability of concrete buildings cured in seawater. Since a few studies investigated concrete properties under the curing condition of Caspian seawater, in order to address this knowledge gap, the present work examined the comparative effects of NA, RHA, and NS on durability and mechanical properties of the concrete under Caspian seawater curing conditions. In addition, the Iranian national building code has several restrictions for construction in the Persian Gulf curing environment in the south of Iran. However, there is a lack of government information and guidelines for building in Caspian Seawater curing conditions. As far as we know, it is the first work studying the impact of these SCMs under the Caspian seawater curing condition. For a comparative assessment of the effect of various SCMs on different properties of concrete, we considered 16 groups of mix designs. With these samples, the effect of different SCMs can be compared because the only feature varying across mix designs with different SCMs that belong to the same category is the kind of SCMs.

## Materials and methods

### Materials

**Figure 1:**
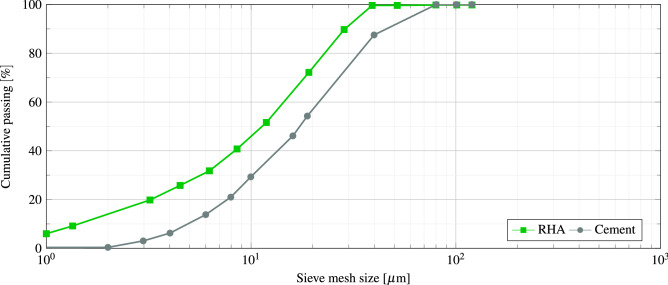
Particle size distribution curves of cement and RHA.

In the present work, a type II ordinary Portland cement is used as the main cementitious binder according to ASTM C 150^59^. The cement producer is Khazar Cement Company (Guilan, Iran). The cement fineness was 3230 $\dfrac{\hbox {g}}{{\hbox {cm}}^2}$ according to ASTM C204-11^60^ and the final and initial setting times were 170 and 110 min, respectively. Besides, the 7 and 28 day compressive strengths and the specific weight of cement were 41 $\left[ \dfrac{\hbox {N}}{{\hbox {mm}}^2}\right]$, 52 $\left[ \dfrac{\hbox {N}}{{\hbox {mm}}^2}\right]$ and 3150 $\left[ \dfrac{\hbox {kg}}{\hbox {cm}^3}\right]$, respectively. The chemical and physical properties of the used cement are given in Tables 1 and 2, respectively. The particle size distribution curve of the cement is shown in Fig. 1.

**Table 1:** The physical characteristics of cement.

Autoclave expansion	0.07	[%]
Specific gravity	3.07	$\left[ \dfrac{{\hbox {g}}}{\hbox {cm}^{3}}\right]$
Specific surface	3120	$\left[ \dfrac{{\hbox {cm}}^2}{\hbox {g}}\right]$
Initial setting time	140	$\left[ \hbox {min}\right]$
Final setting time	190	$\left[ \hbox {min}\right]$

**Table 2:** The chemical characteristics of cement (wt %).

$\hbox {CaO}$	${\hbox {SiO}_{2}}$	${\hbox {Al}_{2}\hbox {O}_{3}}$	${{\hbox {Fe}}_{2}{\hbox {O}}_{3}}$	${{\hbox {SO}}_{3}}$	*MgO*	Free $\hbox {CaO}$	Loss on ignition	${{\hbox {K}}_{2}{\hbox {O}}}$	Insoluble residue	${{\hbox {Na}}_{2}{\hbox {O}}}$
63.7	20.55	4.5	4.35	2.45	1.8	1.46	1.42	0.54	0.47	0.35

**Figure 2:**
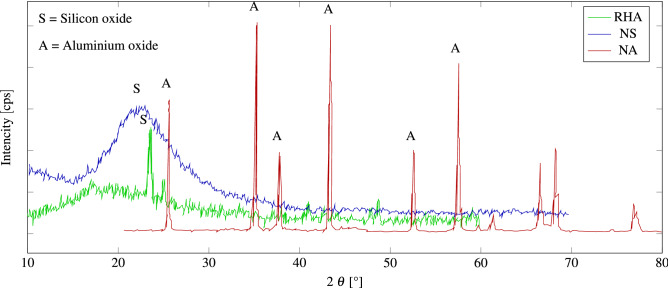
XRD of RHA, NS, and NA.

Rice husk is provided by the rice milling industry since it is a byproduct, burnt for about 72 h in air in an uncontrolled burning process. The temperature range is between 400 and 600 °C. Gray RHA is used in the present study with a pH of 7.3. The size of NS particles with an average specific surface area of 193 $\dfrac{{\hbox {m}}^2}{\hbox {g}}$, 98% purity and particle diameter of 25 nm was prepared by Payazhic Co. Figure 2 indicates powder X-ray diffraction (XRD) diagrams of NS Nanoparticles. NA with the average particle size of 9 nm is utilized without any modification. Table 3 presents the NA properties. Figure 2 indicates powder X-ray diffraction (XRD) diagrams of NS Nanoparticles.

**Table 3:** Characteristics of RHA, NS, and NA.

Composition materials and Physical properties		RHA	NS	NA
${\hbox {SiO}_{2}}$	[%]	90.9	$\geqslant \,$ 98	$\geqslant \,$ 99.8
${{\hbox {Al}_{2}}{\hbox {O}_{3}}}$	[%]	0.83	0.076	–
${{\hbox {Fe}_{2}}{\hbox {O}_{3}}}$	[%]	0.6	0.293	–
$\hbox {CaO}$	[%]	0.8	0.392	–
$\hbox {MgO}$	[%]	0.56	0.05	–
${{\hbox {Na}_{2}}\hbox {O}}$	[%]	1.02	–	–
${{\hbox {K}_{2}}\hbox {O}}$	[%]	3.14	–	–
${{\hbox {Na}_{2}}{\hbox {O}}_{3}}$	[%]		0.328	–
${\hbox {SO}_3}$	[%]	–	0.185	–
${\hbox {TiO}_2}$	[%]	–	0.064	–
${\hbox {P}_{2}{\hbox {O}}_5}$	[%]	–	0.129	–
$\hbox {ZnO}$	[%]	–	0.021	–
$\hbox {CuO}$	[%]	–	0.020	–
Loss on ignition	[%]	2.1	–	–
Loss on drying	[%]	–	< 5	–
Specific gravity	$\left[ \dfrac{\hbox {g}}{\hbox {cm}^3}\right]$	2.13	2.65	
Specific surface	$\left[ \dfrac{\hbox {m}^2}{\hbox {g}}\right]$	0.376	193	205
Bulk density	$\left[ \dfrac{\hbox {kg}}{\hbox {m}^3}\right]$	429.1	50	120
Mean particle size		3.8 $\upmu \hbox {m}$	20–30 nm	8.0–10 nm
PH		6	5–6	3.7–8.5

**Figure 3:**
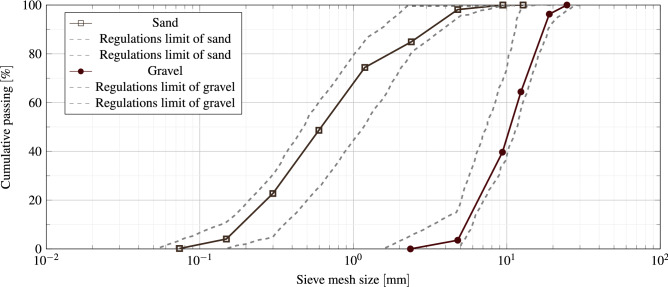
Fine and coarse aggregate grading curves.

Natural river sand with a specific gravity of 2.63, fineness modulus of 2.64, water absorption of 1.3%, bulk density of 1720 $\left[ \dfrac{\hbox {kg}}{\hbox {m}^3}\right]$, and a maximum size of 4.74 mm is employed as fine aggregate. The coarse aggregate is crushed limestone with a specific gravity of 2.56, maximum nominal size of 16 mm, bulk density of 1580 $\left[ \dfrac{\hbox {kg}}{\hbox {m}^3}\right]$, water absorption of 0.78%, and fineness modulus of 6.86. The coarse and fine aggregate gradation curve shows a complete consistency with the ASTM C33 criteria^61^. Figure 3 shows the gradation curve of coarse and fine aggregates with ASTM C33 standard limits.

A Type F polycarboxylate acid-based superplasticizer conforming with ASTM C494^62^ is introduced in all the concrete mixtures as a weight percentage of the total cementitious materials. The superplasticizer was used to help disperse Nanoparticles throughout the concrete mixture, increase compaction, and improve the concrete workability. The characteristics of the superplasticizing admixture materials are given in Table 4.

**Table 4:** Characteristics of superplasticizer additive.

Appearance	Light brown	
Freezing temperatures	About -2	$^\circ$C
Solid content	42	[%]
Specific gravity	1.09	$\left[ \dfrac{\hbox {g}}{\hbox {cm}^3}\right]$

#### Chemical analysis of Caspian seawater

In this study, the water utilized for curing conditions was taken from the Caspian Sea. For determining the chemical properties of Caspian seawater, provided samples were analyzed. Table 5 presents the chemical analysis results. The anions and cations in Caspian Seawater are not found in very high concentrations. Chloride and sulfate with a total quantity of 3894 and 1894 ppm are just two ions somewhat higher than the standards.

**Table 5:** Characteristics of Caspian seawater.

Ion and physical properties	Caspian seawater	Drinking water	ASTM C^63^	
pH	8.54	7.5	5–8.5	
Total dissolved solids	9190	572	2000	[ppm]
$\hbox {Cl}^-$	3987	105	1000	[ppm]
$\hbox {So}^{2-}_{4}$	1894	159	1000	[ppm]

### Concrete mix design and specimen preparation

We cast four series of concrete samples (i.e., PC, NSC, NAC, and RHAC Series). Then, samples are subjected to the experiments. PC signifies plain concrete and is considered as control concrete. The water to binder (the total of Nanoparticles and cement) ratio (W/C) employed for all mixtures is 0.5. RHAC10, RHAC15, and RHAC20 represent the concrete that contain 10, 15, and 20 wt % RHA, by the weight of cement. NSC1, NSC2, NSC3, NSC4, NSC5, NSC6, NSC7, and NSC8 signify the concrete that contain 1, 2, 3, 4, 5, 6, 7, and 8 wt % NS by the weight of cement. NAC1, NAC2, NAC3, and represent the concrete that contain 1, 2, 3, and 4 wt % nano-alumina by the weight of cement. Table 6 represents the mix proportions for one cubic meter of concrete. For producing the concrete containing NPs, we combined superplasticizer with water for 5 min in a mixer prior to the addition of the NPs, and it was stirred at a high speed for another 5 min. After low-speed mixing (2 min) in a concrete centrifugal blender for establishing appropriate workability, the mix of superplasticizer, NPs, and water was added gently, followed by swirling for 2 min at a low speed so that suitable workability is ensured. With dissolving the superplasticizer in water, plain concrete is made. After placement of superplasticizer and water into a concrete centrifugal blender, it is swirled for several minutes so that the optimal workability is obtained prior to the addition of cement, sand, and coarse aggregate.

**Table 6:** Mix proportions of specimens.

Mix No.	Mixture ID	W/C	Water	Cement	RHA	NS	NA	Fine Agg.	Coarse Agg.
			$$\left[ \dfrac{\hbox {kg}}{\hbox {m}^3}\right]$$						
1	PC	0.5	175	350	–	–	–	825	1000
2	RHAC10	0.5	175	315	35	–	–	825	1000
3	RHAC15	0.5	175	297.5	52.5	–	–	825	1000
4	RHAC20	0.5	175	280	70	–	–	825	1000
5	NSC1	0.5	175	346.5	–	3.5	–	825	1000
6	NSC2	0.5	175	343	–	7	–	825	1000
7	NSC3	0.5	175	339.5	–	10.5	–	825	1000
8	NSC4	0.5	175	336	–	14	–	825	1000
9	NSC5	0.5	175	332.5	–	17.5	–	825	1000
10	NSC6	0.5	175	329	–	21	–	825	1000
11	NSC7	0.5	175	325.5	–	24.5	–	825	1000
12	NSC8	0.5	175	322	–	28	–	825	1000
13	NAC1	0.5	175	346.5	–	–	3.5	825	1000
14	NAC2	0.5	175	343	–	–	7	825	1000
15	NAC3	0.5	175	339.5	–	–	10.5	825	1000
16	NAC4	0.5	175	336	–	–	14	825	1000

### Test procedures

#### Workability

**Figure 4:**
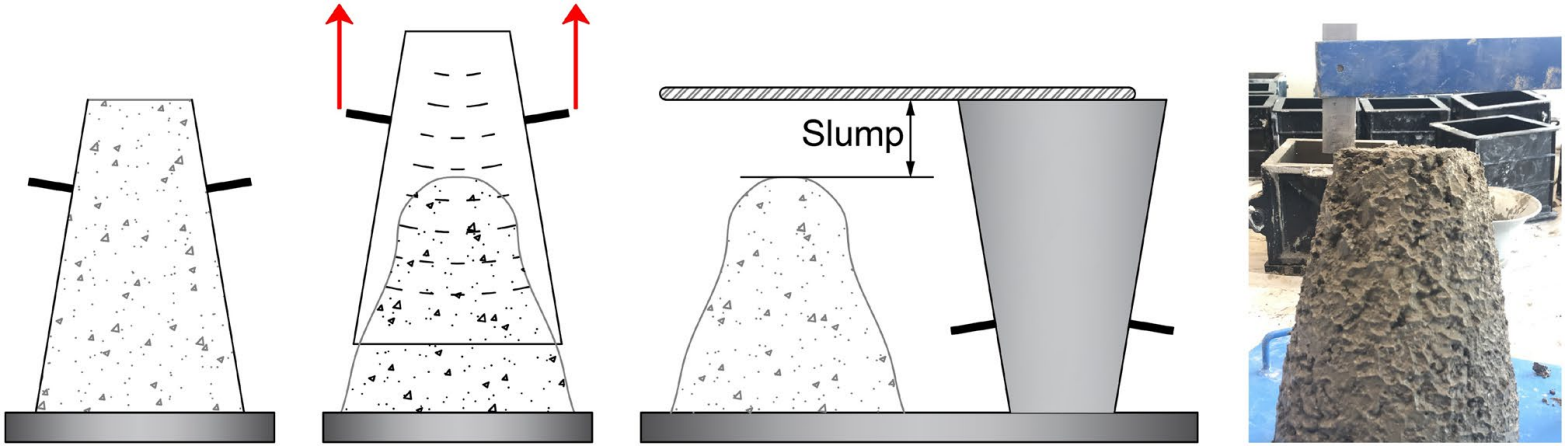
Schematic of slump testing of the concrete mixture.

Standard slump tests are employed in accordance with ASTM C143^64^ for determining the workability of freshly mixed concretes. For the workability test, a big pan, a slump cone (lower diameter: 200 mm, upper diameter: 100 mm, height: 300 mm), a ruler, and a steel tamping rod are utilized. Water was used for cleaning and moistening the internal surface of slump cone Fig. 4. Then, it is placed on a big horizontal smooth pan. New concrete is used for filling the slump cone in four levels, and each layer is about one-fourth of its height, and tamped 25 times using the rounded end of the steel tamping rod. The mold is immediately taken out from the concrete by rising it carefully in the vertical orientation. Lastly, the height difference between the maximum point of the subsided new concrete and maximum point of the slump cone was calculated.

#### Density

For conducting a density test based on ASTM C 642^65^, three samples of concrete specimens were taken out of storage after 28 days of curing and scheduled to be tested on a certain day. After the elimination of water from the specimens’ surface, we transformed them into saturated surface dry (SSD) conditions. Then, the SSD weight of the specimens were specified in the air (C). Afterward, specimens were baked at 100–110 °C for 24 h. The weight of the specimens was then determined that represented their oven-dry weight while suspended in air (A). Later, the samples were placed under water in a bucket, following by determining the weight underwater (D). We also recorded the water temperature on test day (T), and the water density $\rho _w$ at that temperature was specified. The concrete density can be obtained using Eq. (1).

1 $$\begin{aligned} \rho _{\hbox {Concrete}}=\dfrac{{\hbox {A}\rho }_{\hbox {w}}}{(\hbox {C}-\hbox {D})} \end{aligned}$$

#### Compressive strength

**Figure 5:**
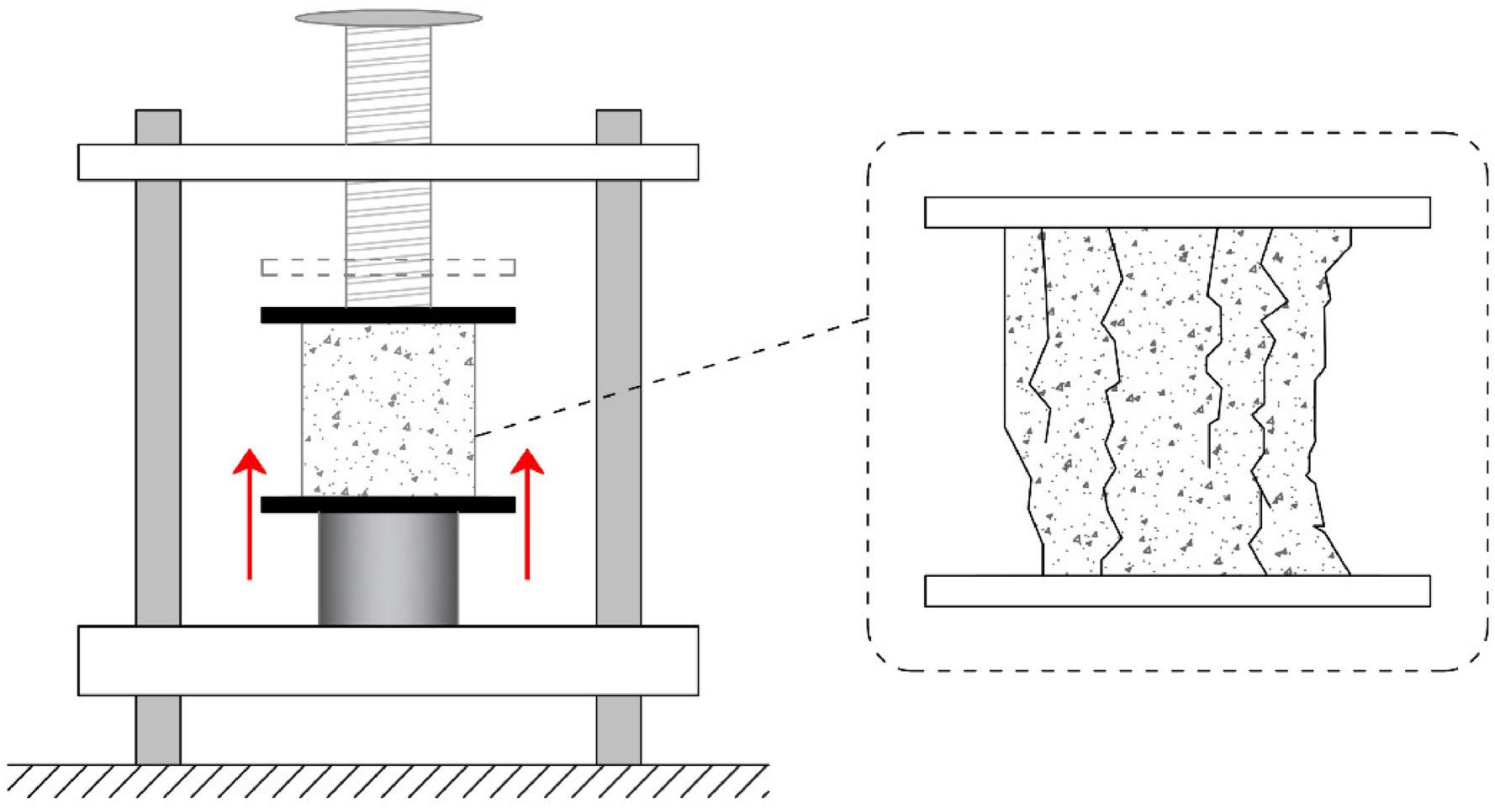
Schematic of compressive strength testing of the concrete mixture.

A 200-ton hydraulic concrete pressure testing machine was used for conducting compressive strength tests in accordance with ASTM C39^66^, as shown in Fig. 5. The samples were loaded at the ages of 7, 14, 28, and 90 days at the rates of 100 $\left[ \dfrac{\hbox {kg}}{\hbox {s}}\right]$ with the force-controlled load application for the compressive strength test. The specimens’ size was 150 mm$\times$ 150 mm$\times$ 150 mm.

#### Water permeability and Chloride ion penetration

**Figure 6:**
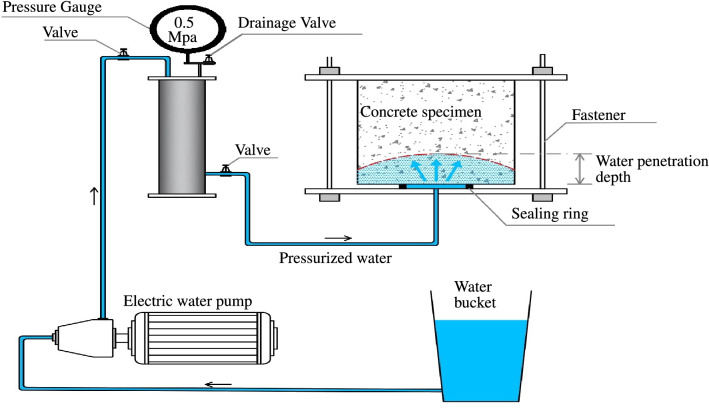
Schematic of penetration testing of the concrete mixture.

Using the automated concrete water penetration system, the water penetration depth into hardened concrete is assessed in three cells according to^67^. Three samples were made (150 mm$\times$ 150 mm$\times$ 150 mm), and then they were coated with wet burlap and a polyethylene sheet for 24 h before demoulding and curing in a water tank for 28 days. We took out samples from curing tank before testing, and they were put in the air for 72 h at 20 °C. Then, samples were placed in the penetration cell for 72 h at a water pressure of 0.5 MPa. The depth of water penetration was calculated by crushing the samples in a compression test machine by the use of the indirect tensile approach. Figure 6 indicates the test setup schematic.

The rapid chloride penetrability of the concrete mixes was determined using a 100 mm diameter and 50 mm thick concrete disk cut from a cylindrical specimen diameter of 100 mm at curing ages of 28 according to ASTM C1202-12^68^. The resistance of concrete to chloride ion penetration is represented by total charge passed in coulombs during a 6 h test.

### Statistical analysis

Each experiment is conducted five times. The mean ± SD is used to represent the results. As a statistical analysis, one-way analysis of variance (ANOVA) is used, followed by the post-hoc Tukey test. *p*-value $\le$ 0.05 was chosen as the criteria for statistical significance in all assessments.

## Results and discussion

### Workability

**Figure 7:**
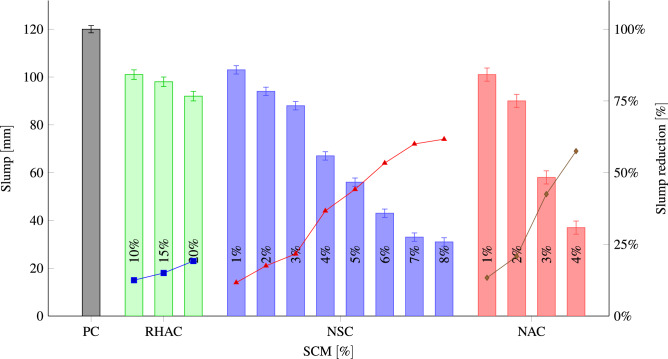
The slump of concrete in each group.

Slump reliably indicates the concrete workability and is utilized for assessing the workability of different mixes of concrete containing the same amount of water. The greater the measured height of slump, the better the workability, which suggests easier flow of the concrete when it is poured. Nevertheless, it is not subjected to segregation at the same time^69^. Figure 7 illustrates the workability test results. It indicates the impact of NA, NS, and RHA content and particle fineness on the workability of mixes while keeping the water to binder ratio constant at 0.50. As demonstrated by the results of the effects of NA, RHA, and NS on the concrete workability, with increasing the percentage substitution of cement with NA, RHA, and NS, concrete gets more unworkable unless we apply water-reducing admixtures. The results indicated significant differences in the slump between PC and RHA ($\hbox {\textit{P}}_{\hbox {value}}<0.047$), NS ($\hbox {\textit{P}}_{\hbox {value}}<0.027$), and NA ($\hbox {\textit{P}}_{\hbox {value}}<0.017$). Similar our results, different studies showed that adding RHA^70^, NS^71–73^ and NA^54,74^ reduced the workability of the concrete. Furthermore, the incorporation of nanoclay^75^ and marble dust powder^76,77^ makes concrete mixtures stiffer, adversely influencing the workability of concrete. In addition, the workability of concrete containing recycled coarse aggregates (RCA) decreased as the percentage of RCA increased^78,79^. RHA has macro and mesoporous particles and their specific surface area increases with fineness. Water is absorbed by the surface of fine RHA and it is stored in its pores, which reduces free water and slump value^32^. Likewise, the increased reactivity of RHA could be another factor that contributes to the decrease in the concrete flow^80^. Specific area of NA and NS is larger than cement, which leads to greater water absorption and decreases the concrete slump. Also, NS has numerous unsaturated bonds and a large specific surface area that makes it simpler to attract water molecules for forming chemical bonds^81^. The low dosage of the NA $( \geqslant 3)$ and NS $( \geqslant 5)$ provides suitable workability. With the extreme reduction in slump and high plastic viscosity, molding challenges is made, and heterogeneous casting can cause undesired properties, which may be worrying.

### Compressive strength

Table 7 presents the compressive strength values achieved for different concrete specimens with various NA, RHA, and NS replacement percentages. The samples’ compressive strength showed an improvement with increasing age to the extent that the strength reached a peak at 90 days. The best performing mixtures of the RHA, NS, and NA are those containing 10% ($\hbox {\textit{P}}_{\hbox {value}}<0.03$), 6% ($\hbox {\textit{P}}_{\hbox {value}}<0.003$), and 3% ($\hbox {\textit{P}}_{\hbox {value}}<0.02$), respectively. The mixtures with the best performance of the NA, RHA, and NS are those that contain 1, 10, and 6%. As indicated by the comparison of RHA results, compressive strength enhances when RHA is employed in favor of cement up to a 10% partial replacement and then decreases. Nevertheless, samples with a 15% replacement level have still the same compressive strength as PC specimens. When the cement is replaced by 20% RHA, compressive strength is lowered, reducing to a value below the value of PC. Similar findings were reported by Ganesan et al.^82^, who found that using adding 15% of RHA as cement replacement showed an increase by 26% of compressive strength at 28 days of curing. While 20% of RHA replacement showed equivalent in strength at 56 and 91 days of curing respectively. It can be because the RHA amount in the mix is higher than the amount needed for combining with the released lime during the hydration process, which results in excessive leaching of silica. Thus, it leads to a lack of strength as it replaces some of the cementitious material but has no contribution to strength^83^. Compressive strength is reduced for early-age concrete samples at 7 days in the presence of RHA in comparison with control concrete. The reason for such reduction in early-age strength is lower pozzolanic activity of RHA as an outcome of its coarser particles. Besides, it is likely that the reduced volume of hydration products related to a less favorable hydration rate leads to a decrease in early-age strength^84^. Studies^85,86^ have shown that even a small percentage of NS added to concrete while it is early age may dramatically increase the compressive strength. The improved compressive strength of NSCs at an early age is associated with the NS’s strong pozzolanic activity at an early age, while the improvement in 28-day compressive strength can be attributed to the C-S-H gel filling up voids and improved mortar-aggregated bonding. The space between the grains in the cement paste is filled by finer and smaller NS particles, which results in increasing the strength^87^. The compressive strength increases up to 2.0% NA replacements, then decreases, while the addition of 4.0% NA leads to samples with similar compressive strength, like CP. The reduction of compressive strength due to the addition of more than 2 % NA and 6.0% NS could be affected by the fact that the of NA and NS injected into the mix is more than the amount required for the combination with the freed lime during the hydration process. Besides, it can be associated with faults in the dispersion of NPs, forming weak zones. Heikal et al.^88^found that adding 1% NA to cement enhanced compressive strength by 30.67%. Similarly, Nazari and Riahi^89^ showed that the compressive strength of mortar could be significantly improved by introducing NA.

The RHA, NS, and NA may have a similar filler effect. However, NS is a very reactive pozzolanic material from a chemical viewpoint. When cement is hydrated, it combines with calcium hydroxide (CH) to create calcium silicate hydrate (C–S–H). The NS’s large specific surface area will accelerate cement hydration and pozzolanic reaction. Before the concrete samples were made for 24 h, the NS’s pozzolanic reaction might have begun, enhancing the early strengths. It’s possible that NS served as a reactive filler, which takes up space between cement and slag particles to stop bleeding and boost packing density of solid components. Given its size and form, RHA causes early-stage samples to exhibit delayed pozzolanic activity. The compressive strength of concrete mixes containing recycled coarse aggregate and natural coarse aggregate decreases as the percentage of RHA increases^90^, which is consistent with our findings for RHA. Similar to NS and NA, adding copper slag up to a certain percentage increases the compressive strength; after that, the compressive strength decreases^91^. Furthermore, replacing natural coarse aggregate with polypropylene^92^ and polyethylene terephthalate^93^ decreases the compressive strength of concrete.

**Table 7:** Changing compressive strengths of concrete containing RHA, NS, and NA at various ages.

Mix No.	Mixture ID	Compressive strength $\left[ \dfrac{\hbox {N}}{{\hbox {mm}}^2}\right]$			
		7 days	14 days	28 days	90 days
1	PC	19.3 ± 0.7	23.2 ± 0.4	32.2 ± 0.6	35.2 ± 0.8
2	RHAC10	17.7 ± 0.5	24.8 ± 0.2	30.45 ± 0.4	38.3 ± 0.4
3	RHAC15	16.6 ± 0.9	24.1 ± 0.5	28.3 ± 0.9	35.4 ± 0.3
4	RHAC20	15.1 ± 0.3	23.1 ± 0.7	27.9 ± 0.7	34.9 ± 0.4
5	NSC1	19.8 ± 0.5	24.1 ± 0.1	29.2 ± 0.4	37.1 ± 0.4
6	NSC2	20.5 ± 0.7	25.3 ± 0.8	30.5 ± 0.8	38.6 ± 0.6
7	NSC3	21.5 ± 0.8	26.5 ± 0.6	31.8 ± 0.6	40.1 ± 0.3
8	NSC4	22.7 ± 0.4	27.4 ± 0.7	33.2 ± 1.1	41.5 ± 0.5
9	NSC5	23.6 ± 0.4	28.1 ± 0.8	34.2 ± 0.8	43.3 ± 0.7
10	NSC6	24.3 ± 0.6	28.5 ± 0.7	34.9 ± 1	45.2 ± 0.8
11	NSC7	23.4 ± 0.7	27.9 ± 0.6	34.3 ± 0.6	43.1 ± 0.6
12	NSC8	21.1 ± 0.9	26.2 ± 0.9	33.1 ± 0.5	40.2 ± 0.4
13	NAC1	19.6 ± 0.4	24.7 ± 0.4	28.2 ± 0.3	35.9 ± 0.7
14	NAC2	20.7 ± 0.3	26.5 ± 0.9	29.8 ± 0.5	37.6 ± 0.6
15	NAC3	19.9 ± 0.6	27.2 ± 0.7	29.7 ± 0.4	38.5 ± 0.4
16	NAC4	19.6 ± 0.2	26.1 ± 0.4	27.9 ± 0.8	35.4 ± 0.6

### Density

**Figure 8:**
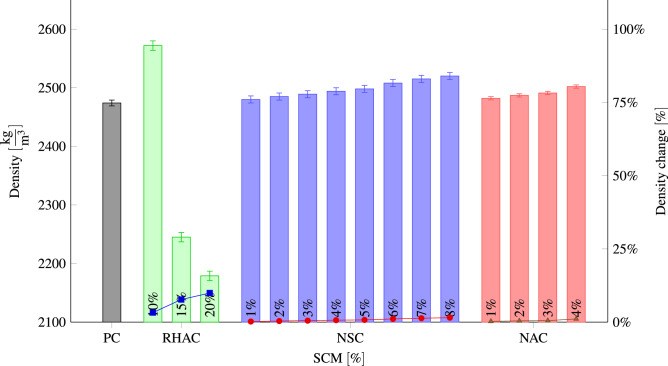
Density for different percentages of RHA, NS, and NA replacement.

Figure 8 indicates the concrete density in detail, obtained using concrete cubic samples that contain different percentages of NA, RHA, and NS. The concrete density improves with replacing cement by RHA particles because of its high pozzolanity, pore refinement, and filler action. Ziad et al.^94^ also showed that by adding 5%, 10%, 15%, and 20% RHA the density of concrete decreased. The increasing density of concrete is affected by amorphous silica concentration, fineness, and RHA replacement level. The pozzolanic activity and slower hydration of RHA blended cement can rationalize the loss in density of concrete above the 10% replacement. Due to the cement compatibility and high pozzolanic of RHA, a decline in the portlandite content of the matrix and an increase in the quantity of C S H products can result in improved pore structure of the concrete matrix^95^. With the replacement of the cement with NA and NS, the density is increased, which can be attributed to the cement paste hydration in particular. Hydration products become increasingly interconnected and compact as a unit. In comparison with the early age, the overall porosity showed a dramatic reduction. The small particle size of NA and NS has a filling impact, and they fill the space between un hydrated particles and voids from thicker cement paste, which lowers the the cement paste’s overall porosity.

**Figure 9:**
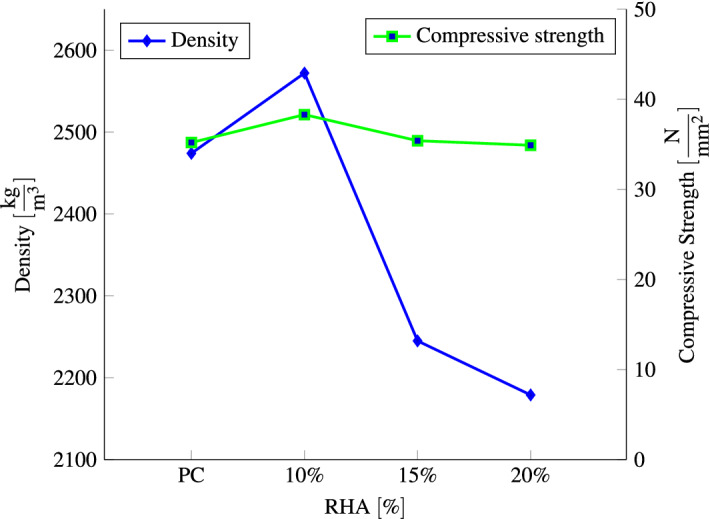
Relationship between compressive strength and density after 28 days for different percentages of RHA.

The relationship between compressive strength and density, as well as the effect of RHA content is shown in Fig. 9. Based on the relationship, the compressive strength and density of concrete increases with the increasing of the RHA content ratio until 10% and and then both decreased with adding 15% and 20% of RHA. The concrete achieves the highest compressive strength of 38.3 MPa with 2572 $\frac{\hbox {kg}}{\hbox {m}^3}$ as compared to others.

Several studies came to the same results as we did, namely that adding NS^96–98^ and NA^50,99^ resulted in an increase in concrete density. Furthermore, the incorporation of nano${\hbox {TiO}}_2$^17^, copper slag^91^ and marble dust^75^ increase the concrete density . In contrast to NS and NA, increasing amounts of waste expanded polystyrene^100^ and ethylene-vinyl acetate^101^ are added to concrete, resulting in a significant decrease in density. Similar to our results, Behera and Rahman showed that adding RHA decreased concrete density^102^.

### Water permeability and chloride ion penetration

**Figure 10:**
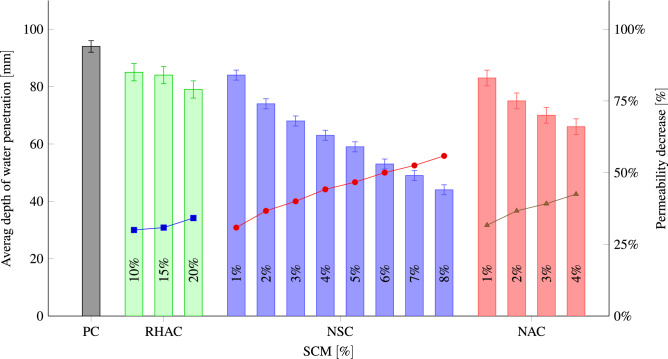
Average depth of water penetration at 28 days for different percentages of RHA, NS, NA replacement.

**Figure 11:**
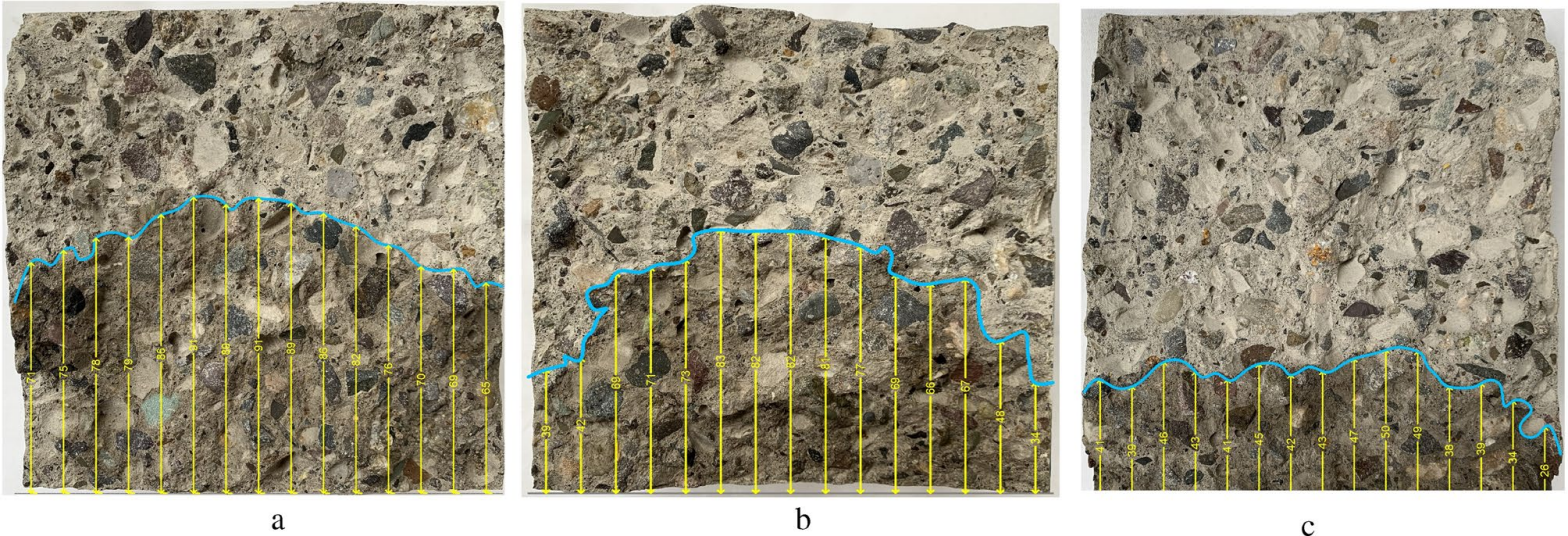
Water penetration area and penetration front in (**a**) RHAC20, (**b**) NSC8, and (**c**) NAC4.

Figure 10 shows the permeability test results. The depth of water penetration reduced with increasing percentage of NA, RHA, and NS, and the density trend is followed. Permeability is reduced by replacing the cement with NA, RHA, and NS due to their specific surface area that is greater than the replaced cement. It enhances the absorption of the free water present in the mixing of the desired concrete. Besides, in concrete comprising NA, RHA, and NS, the thickness of the interfacial transition zone that around the coarser size aggregate is small in comparison with the PC. Water penetration area and penetration front in RHA20, NS8, and NA4 are demonstrated in Fig. 11. Iskra–Kozak et al.^103^ and Rezania et al.^71^ showed that adding 1–4% NA similarly reduced water permeability in concrete.

**Figure 12:**
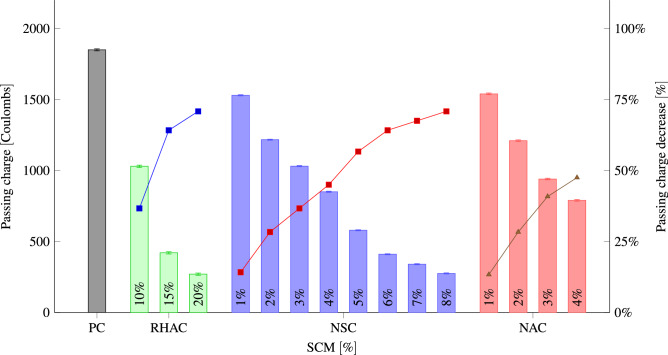
Rapid chloride penetrability at 28 days for different percentages of RHA, NS, NA replacement.

Figure 12 indicates the average passing charges and penetration depths for three replicate samples. It also indicates qualitative evaluation for the penetrability of the chloride ions. With increasing the replacement level, the charge passed reduces for all SCMs. The high dosage of RHA (10% and 15%), NS (3–8%), and NA (3% and 4%) might decline the rapid chloride penetrability of concrete from a low rating to very low ratings from higher to lower replacement levels. With an increase in NA, RHA, and NS content and fineness, concrete pore structure can be made finer and the volume of big pores and overall porosity in the concrete can be reduced. Therefore, an increase in the percentage of NA, RHA, and NS reduces the depth of water and chloride ion penetration. Al Mamun and Islam^104^ reported the same decrease in the penetrability of the chloride ions with the increase in RHA dosage.

**Figure 13:**
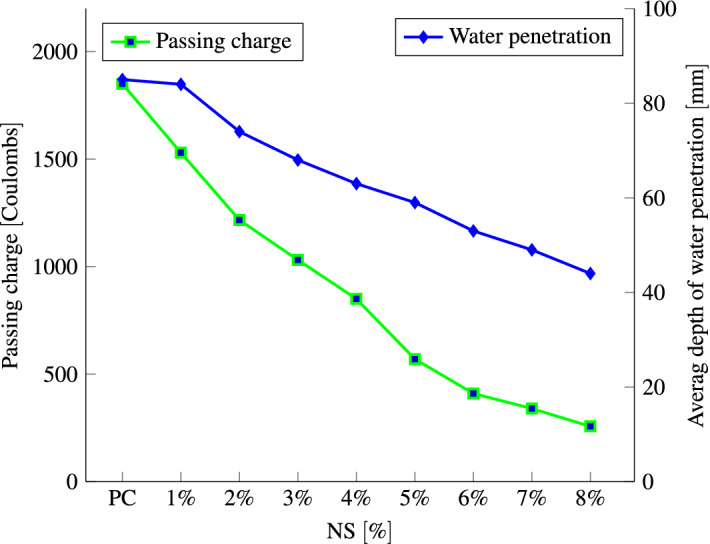
Relationship between the chloride penetrability and water penetration after 28 days for different percentages of NS.

The relationship between chloride penetrability and water penetration, as well as the effect of NS content is shown in Fig. 13. Based on the relationship, the chloride penetrability and water penetration of concrete decreased with the increasing of the NS content ratio. It revealed that the chloride penetrability is directly proportional to NS content and water penetration. The concrete achieves the the minimum water penetration of 44 mm with 275 Coulombs passing charge with adding 8% NS. Similar to our findings, studies have demonstrated that adding RHA^95,105–107^, NS^41,108–110^, and NA^103,111^ to concrete reduces chloride ion penetration and water absorption. Furthermore, adding nano$\hbox {TiO}_2$ to RHA-enriched cement mortars improved the composite’s chloride resistance^112^, while adding polyethylene terephthalate aggregates increases the depth of water penetration^113^. Moreover, adding of fly ash and coal gangue decreased the chloride diffusion coefficient^114^.

## Conclusions

In the present study, the effect of incorporating RHA and two NPs (nano-aluminum and nano-silica) in the Caspian Sea curing conditions on the durability and mechanical behavior of the concrete was analyzed. These materials were investigated concurrently by performing slump, density, compressive, chloride ion penetration, and permeability tests. As conclusion:

- According to results of Fresh properties, workability reduced with increasing amount of NA, RHA, and NS. The consistency of mortars is affected by the addition of NA, RHA, and NS, and NA as a partial replacement for cement. With an increase in the content of these SCMs, the water demand for the binder is also increased. Lowest workability was observed in the mix comprising 8% NS. NA, RHA, and NS have a large specific surface area because of their small particle size. In the process of concrete mixing, many unsaturated bonds improve the NA, RHA, and NS for absorbing more water molecules, leading to the reduction of concrete slump.
- In comparison with NA and RHA, the NS addition caused a significant increase in compressive strength in the early phases. Nevertheless, after 14 days, no noticeable difference was noted in the performance of concrete that contained NA, RHA, and NS. The highest compressive strength of all samples was observed in NSC6, followed by NSC5.
- With introducing RHA 10%, NS, and NA the concrete density increased. The increase in density is consistent with the results of depth of water and chloride ion penetration. The maximum increase in density was achieved by RHAC10, with an increase of 3.8% in comparison with the control sample.
- when cement was replaced by NA ($\geqslant 3 \ \%$) and NS ($\geqslant 4 \ \%$), a marked rise in penetration depth was noted after 28 days. Besides, these samples showed better mechanical performance. The best result was obtained by NSC8, giving a decrease of 53% compared to the control sample.
- Cement mixes made with NA, RHA, and NS decreased the chloride ion penetrability. The charge passed was reduced by increasing the replacement. Very low permeability was obtained by the 15% RHA, 20 % RHA, NS 3–8%, and NA 4% replacement to cement, and moderate permeability was recorded for the control mix and other mix designs. Among all samples, the highest chloride permeability improvement was noticed with NSC6, NSC7, and NSC8, among which NSC8 had the lowest chloride permeability.
- Comparison of the tested properties indicated that NSC8 presented the best all-round performance.
- This research aims to find a solution to improve the mechanical properties and durability of concrete cured with the Caspian Seawater. Future research on the impact of sea-level fluctuations on concrete structures and also frost resistance, which are significant components that might influence the lifespan of structural concrete, appears prudent.

## Data Availability

The datasets that were generated and/or analysed during the current study are freely available from the corresponding author on a request.
